# Impact of Synbiotics Intake on Body-Composition-Related Indicators and Physical Performance in Judokas Before the Match

**DOI:** 10.3390/nu18101503

**Published:** 2026-05-08

**Authors:** Tomomi Yoshikawa, Yukihiro Yokoyama, Takahiro Kuno, Yuji Nimura, Hidetoshi Matsunami

**Affiliations:** 1 Matsunami Research Park, Sosaikouseikai Clinical Foundation, Kasamatsu 501-6062, Gifu, Japan; hide-matsunami@retriever.jp; 2 Department of Perioperative Medicine, Aichi Medical University, Nagakute 480-1195, Aichi, Japan; hiroyoko1208@gmail.com; 3 Division of Surgical Oncology, Department of Surgery, Nagoya University Graduate School of Medicine, Nagoya 466-8550, Aichi, Japan; 4 Aichi University Judo Club, Nagoya 453-8777, Aichi, Japan; tkuno@vega.aichi-u.ac.jp; 5 Aichi Cancer Center, Nagoya 464-8681, Aichi, Japan; y-nimura@tbu.t-com.ne.jp; 6 Nagoya University Judo Club, Nagoya 464-8601, Aichi, Japan; 7 Matsunami General Hospital Judo Club, Kasamatsu 501-6062, Gifu, Japan; 8 Department of Surgery, Matsunami General Hospital, Sosaikouseikai Clinical Foundation, Kasamatsu 501-6062, Gifu, Japan

**Keywords:** synbiotics, physical performance, fecal succinic acid, rebound jump power

## Abstract

**Background/Objectives:** This study aimed to investigate the effects of synbiotics intake on body-composition-related indicators and physical performance in judokas from 4 weeks to 3 days before the match. The associations between the changes in fecal succinic acid concentration and the rate of change in physical performance were also examined. **Methods:** A total of 16 male participants from Aichi University Judo Club were included in a repeated-measures design to compare the effects of non-synbiotics and synbiotics. Body-composition-related indicators and physical performance were assessed, and fecal samples were collected 4 weeks and 3 days before the match. **Results:** From 4 weeks to 3 days before the match, no significant changes in body composition were observed in either the non-synbiotics or synbiotics group. Regarding physical performance, the non-synbiotics group demonstrated a significant reduction in countermovement jump and rebound jump power. In contrast, the synbiotics group maintained physical performance without significant changes. In addition, the change in fecal succinic acid concentration in the synbiotics group was negatively associated with the rate of change in physical performance, particularly rebound jump power (*p* = 0.052). In the rebound jump power-decreased group, the change in the fecal succinic acid concentration increased, whereas it decreased in the rebound jump power-increased group, and the difference between the two groups was statistically significant (*p* = 0.024; AUC = 0.84). **Conclusions:** Before the match, synbiotics intake and a reduction in the fecal succinic acid concentration may be associated with the maintenance of physical performance in judokas, which may have implications for match performance.

## 1. Introduction

In recent years, there has been growing recognition among athletes and coaches that enhancing match performance requires not only the development of technical skills but also the optimization of conditioning before the match [1,2]. Even a judoka who exhibits high levels of skill during practice may struggle to replicate the same level of performance during the actual match. This is because physical factors, such as muscle strength and reaction speed, play a crucial role in technical execution and tactical decision-making during the match [3]. If these factors are not effectively utilized, it may result in a decline in match performance [4].

Judo is a weight-class sport, and it is common for a judoka to lose weight before the match. In particular, rapid weight loss [5] has been reported to have detrimental effects on the maintenance of muscle mass, physical strength, and overall athletic performance [6,7,8]. Previous studies have primarily focused on rapid weight loss immediately before the match [7,9,10]. However, the changes in body composition and physical performance that occur during the weight-loss process over several weeks before the match remain insufficiently investigated.

In our previous study, we found that higher fecal succinic acid levels before Tabata-style high-intensity interval training (T-HIIT) were associated with reduced improvements in physical performance, indicating a negative association between fecal succinic acid concentration and physical performance improvement [11]. Previous studies in patients undergoing gastrointestinal surgery have reported that perioperative administration of synbiotics prevents bacterial translocation and reduces the incidence of postoperative infectious complications [12,13,14,15,16]. Additionally, studies involving patients with sepsis have reported that fecal succinic acid concentrations were lower in the synbiotics intake group than in the non-synbiotics group [17]. However, the association between the synbiotics intake and alterations in body composition-related indicators, physical performance, and fecal succinic acid concentrations in judokas before the match has yet to be elucidated.

The present study aimed to examine changes in body composition-related indicators and physical performance in judokas from 4 weeks to 3 days before the match. Furthermore, the effects of synbiotics intake on these changes were evaluated. Additionally, the associations between the changes in fecal succinic acid concentration and physical performance were also investigated.

## 2. Materials and Methods

### 2.1. Participants and Study Design

This study was conducted for 4 weeks in July–August 2024 and July–August 2025 prior to the Tokai Student Judo Weight Class Championships (an annual, in-a-row tournament). A total of 19 judokas from the Aichi University Judo Club were initially recruited, 3 of whom were excluded due to injury, resulting in 16 participants (aged 18–22 years) being included in the final analysis. All participants were in good physical condition at the time of data collection. A repeated-measures study design was employed to facilitate within-subject comparisons. Participants were assigned to the non-synbiotics group before the 2024 match and to the synbiotics group before the 2025 match. Since both study periods were conducted during the same season and the dietary habits of the judokas remained largely unchanged, the study was carried out under conditions that minimized potential seasonal and dietary effects. However, the training load was not quantitatively monitored, although all participants belonged to the same team and followed a common training program during the study period. Dietary intake was not strictly controlled or recorded, and participants were instructed to maintain their habitual eating patterns. Hydration status and weight-cutting strategies were also not quantified, and participants followed their usual pre-competition routines.

### 2.2. Protocol for Synbiotics Intake

A synbiotics preparation, Synprotec^®^ (Yakult Honsha, Tokyo, Japan), was used in this study. Synprotec contains *Bifidobacterium breve* strain Yakult (1 × 10^8^ CFU/g) and *Lactobacillus casei* strain Shirota (1 × 10^8^ CFU/g) as probiotic components, along with galactooligosaccharides as prebiotics. The judokas were instructed to consume two sets per day (one set consisting of two sachets) for 4 weeks, providing a total daily intake of 2 g of probiotics and 13.6 g of galactooligosaccharides. To monitor intake compliance, each participant installed a dedicated smartphone application (Preha; Yakult Honsha, Tokyo, Japan) and recorded synbiotics consumption at each administration. Data entries were remotely checked, and participants were reminded by the coach when records were missing.

### 2.3. Assessment of Body-Composition-Related Indicators and Physical Performance

Body-composition-related indicators and physical performance were measured at 4 weeks and 3 days before the match, respectively. Body weight (BW) and body composition indicators, including muscle mass (MM), body fat percentage (BF%), and the muscle-mass-to-fat-mass ratio (MM/FM ratio), were assessed. The squat jump (SJ), countermovement jump (CMJ), and rebound jump (RJ) were employed as physical performance measures. For the SJ, participants began from a static position with the knees flexed to 90° and performed a maximal vertical jump without any countermovement. They were instructed to keep the lower limbs extended during takeoff. For the CMJ, participants started from an upright standing position, rapidly descended until the knee joint angle reached 90°, and immediately performed a maximal vertical jump. For the RJ, participants stood upright with their hands on their hips and, upon a signal, performed six consecutive maximal jumps in place, aiming to minimize ground contact time. They were instructed to maximize jump height while achieving rapid takeoffs. Ground contact was made with the forefoot, and heel contact with the floor was not permitted. Jump height and ground contact time were recorded. Rebound jump power (RJ power) and the rebound jump index (RJ index) were automatically calculated from jump height, ground contact time, and body weight using analysis software (Four Assist Ver. 1.10d and Ver. 1.13, Tokyo, Japan). Each test was performed twice, and the higher value was used for statistical analysis. This procedure was adopted to reduce participant fatigue and testing burden. Sufficient rest was provided between trials to ensure measurement reliability. Previous studies have reported that acceptable reliability can be obtained with two to three trials in jump performance tests [18,19].

The body composition was assessed using a multi-frequency bioelectrical impedance analyzer (DC-320; Tanita Corp., Tokyo, Japan). The jump performance, including the squat jump (SJ), countermovement jump (CMJ), and rebound jump (RJ), was measured using a multi-time measurement system (FMT-TC03J, Four Assist, Tokyo, Japan), which records flight time (and contact time for RJ) and calculates jump height based on standard kinematic equations.

### 2.4. Fecal Sample Collection

Approximately 0.2 g of feces was collected from each judoka using a Metabolo Keeper collection kit (Techno Suruga Laboratory Co., Ltd., Shizuoka, Japan) to determine the fecal succinic acid concentrations at 4 weeks and 3 days before the match, respectively.

### 2.5. Measurement of Succinic Acid Concentration in Feces

The fecal concentration of succinic acid was measured by Techno Suruga Laboratory Co., Ltd., using pH-buffered post-column conductivity detection and is expressed as milligrams per gram of feces.

### 2.6. Statistical Analysis

Statistical analyses were performed using JMP software (version 18.1.2; SAS Institute, Cary, NC, USA). Continuous variables are presented as mean ± standard deviation (SD) or median with interquartile range (IQR), as appropriate according to data distribution. Paired comparisons were conducted using the paired *t*-test or the Wilcoxon signed-rank test, and comparisons between the independent groups were performed using the Wilcoxon rank-sum test. Effect sizes were calculated as Cohen’s d using pooled standard deviations, and 95% confidence intervals (CIs) were estimated. Correlations were assessed using Spearman’s rank correlation coefficient. The area under the receiver-operating characteristic (ROC) curve (AUC) was calculated, and the significance of the model was evaluated using logistic regression analysis. A *p*-value of less than 0.05 was considered statistically significant. Multiple comparisons were performed without formal adjustment; therefore, the results should be interpreted with caution due to the potential for increased type I error.

## 3. Results

### 3.1. Characteristics of Judokas

Table 1 summarizes the baseline characteristics of the non-synbiotics group and the synbiotics group. Both groups did not differ in terms of baseline characteristics within participants.

### 3.2. Body-Composition-Related Indicators from 4 Weeks to 3 Days Before the Match in the Non-Synbiotics and Synbiotics Groups

**Figure 1:**
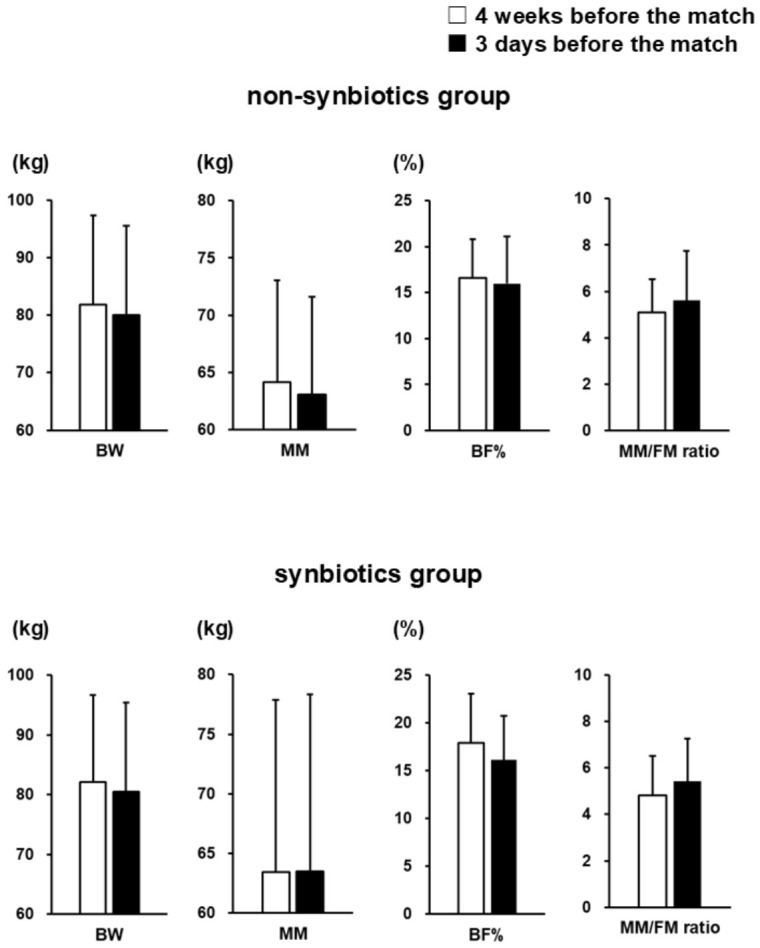
Body-composition-related indicators 4 weeks and 3 days before the match in the non-synbiotics and synbiotics groups. Data are shown as mean ± SD. BW, body weight; MM, muscle mass; BF%, body fat percentage; MM/FM ratio, muscle-mass-to-fat-mass ratio. No significant differences were observed between the non-synbiotics and synbiotics groups.

In the non-synbiotics group and the synbiotics group, no significant changes from 4 weeks to 3 days before the match were observed in mean BW, MM, BF%, and MM/FM ratio (Figure 1).

### 3.3. Physical Performance Change from 4 Weeks to 3 Days Before the Match in the Non-Synbiotics and Synbiotics Groups

**Figure 2:**
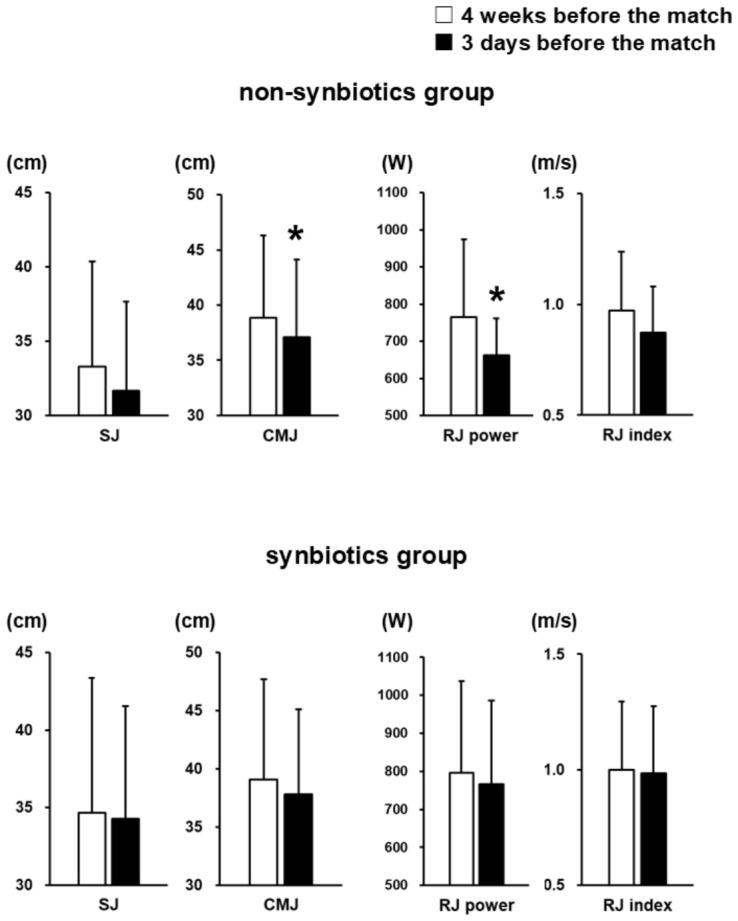
Physical performance 4 weeks and 3 days before the match in the non-synbiotics and synbiotics groups. Data are shown as mean ± SD. * *p* < 0.05 by paired *t*-test. SJ, squat jump; CMJ, countermovement jump; RJ power, rebound jump power; RJ index, rebound jump index. CMJ and RJ power significantly decreased only in the non-synbiotics group.

In the non-synbiotics group, the mean CMJ and RJ power significantly decreased by 4.6% and 13.3%, respectively, from 4 weeks to 3 days before the match (CMJ, from 38.9 ± 7.4 to 37.1 ± 7.0 cm, *p* = 0.022, 95% CI [−3.2, −0.3], d = −0.64; RJ power, from 764.1 ± 209.0 to 662.8 ± 98.1 W, *p* = 0.049, 95% CI [−202.2, −0.3], d = −0.53). Other physical performance also showed a decreasing trend before the match. In contrast, in the synbiotics group, physical performance indexes showed a slight decreasing trend without significant changes (Figure 2).

### 3.4. Comparison of Rate of Change Between the Non-Synbiotics and Synbiotics Groups

**Figure 3:**
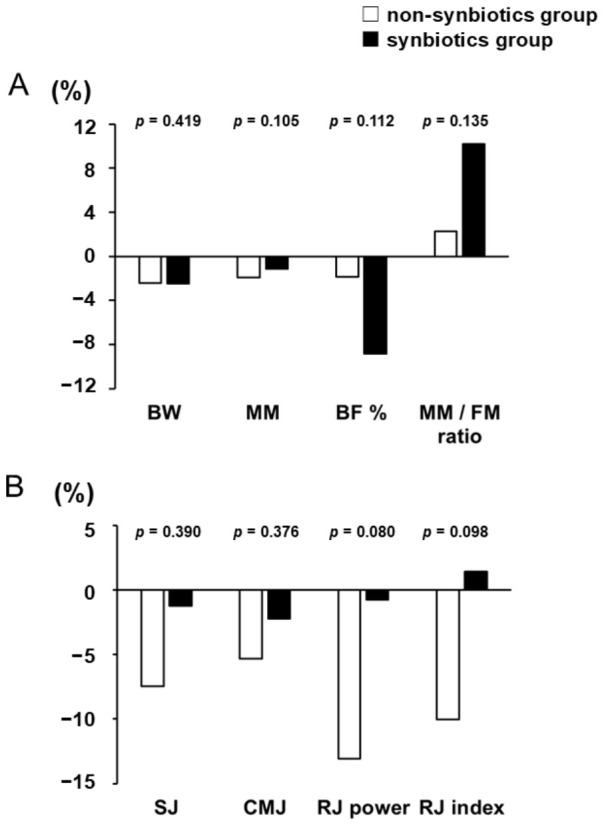
(**A**) The rate of change in body-composition-related indicators between the non-synbiotics group and the synbiotics group from 4 weeks to 3 days before the match. The rate of change in body composition tended to differ between groups, with a greater decrease in MM in the non-synbiotics group and greater decreases in BF% and increases in the MM/FM ratio in the synbiotics group. (**B**) The rate of change in physical performance between the non-synbiotics group and the synbiotics group from 4 weeks to 3 days before the match. Data are shown as median. *P*-values for each comparison are calculated using the Wilcoxon signed-rank test. The rate of change in physical performance, particularly in RJ power and RJ index, tended to show greater declines in the non-synbiotics group compared with the synbiotics group.

From 4 weeks to 3 days before the match, the median rate of change in BW was similar between the non-synbiotics and synbiotics groups. Compared with the synbiotics group, the non-synbiotics group showed a greater decrease in MM (*p* = 0.105), whereas the synbiotics group showed a greater decrease in BF% (*p* = 0.112) and a greater increase in the MM/FM ratio (*p* = 0.135) (Figure 3A).

Furthermore, the non-synbiotics group demonstrated a greater decrease in the median rate of change in physical performance from 4 weeks to 3 days before the match, particularly for RJ power (−13.1% [IQR: −20.0 to 4.5] in the non-synbiotics group vs. −0.7% [IQR: −11.1 to 8.3] in the synbiotics group, *p* = 0.080) and RJ index (−10.0% [IQR: −20.4 to 7.4] in the non-synbiotics group vs. +1.5% [IQR: −10.0 to 9.9] in the synbiotics group, *p* = 0.098) (Figure 3B).

### 3.5. Change in Fecal Succinic Acid Concentration from 4 Weeks to 3 Days Before the Match in the Non-Synbiotics and Synbiotics Groups

**Figure 4:**
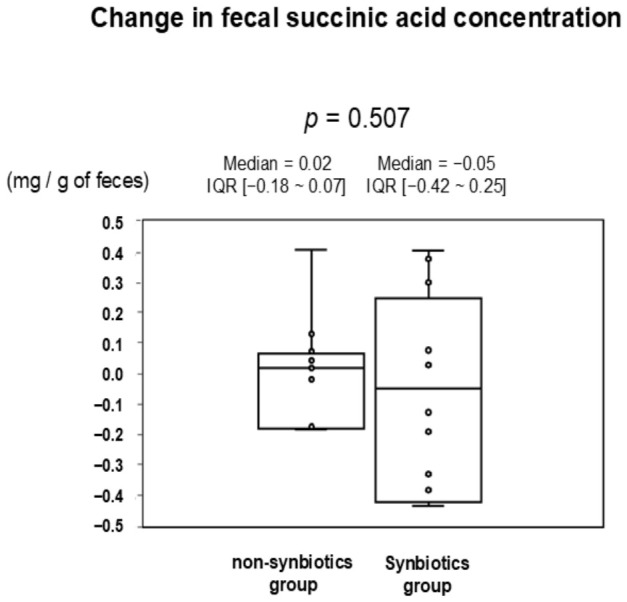
Change in fecal succinic acid concentration between the non-synbiotics group and synbiotics group from 4 weeks to 3 days before the match. Data are shown as median (interquartile range) using box-and-whisker plots. *p*-values are calculated using the Wilcoxon signed-rank test. The median change in fecal succinic acid concentration increased in the non-synbiotics group and decreased in the synbiotics group, with no significant between-group difference.

The median of change in fecal succinic acid concentration showed an increase in the non-synbiotics group and a decrease in the synbiotics group from 4 weeks to 3 days before the match, but no statistically significant difference was observed between groups (Figure 4).

### 3.6. Correlation Between the Change in Fecal Succinic Acid Concentration and the Rate of Change in Physical Performance from 4 Weeks to 3 Days Before the Match in the Synbiotics Group

**Figure 5:**
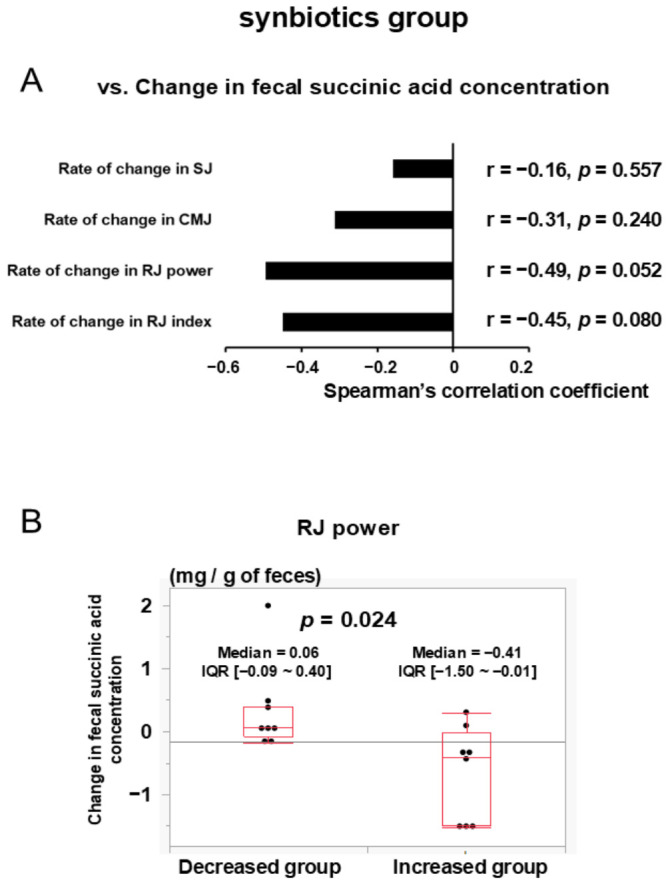
(**A**) Correlation between the change in fecal succinic acid concentration and the rate of change in physical performance from 4 weeks to 3 days before the match in the synbiotics group. *p*-values are calculated using Spearman’s rank correlation coefficient. (**B**) Box-and-whisker plots showing the change in fecal succinic acid concentration in the synbiotics group. Group differences were assessed using the Wilcoxon rank-sum test. In the synbiotics group, rates of change in physical performance were negatively correlated with changes in fecal succinic acid concentration, and the change in fecal succinic acid concentration differed significantly between participants with increased and decreased RJ power.

In the synbiotics group, all physical performance index changes showed negative correlations with the change in fecal succinic acid concentration from 4 weeks to 3 days before the match (Figure 5A). No such correlations were observed in the non-synbiotics group.

### 3.7. Change in Fecal Succinic Acid Concentration from 4 Weeks to 3 Days Before the Match and Its Association with RJ Power-Decreased/-Increased Group in the Synbiotics Group

From 4 weeks to 3 days before the match, in the synbiotics group, the change in fecal succinic acid concentration increased in the RJ power-decreased group (median change = 0.06 mg/g of feces [IQR: −0.09 to 0.40]) and decreased in the RJ power-increased group (median change = −0.41 mg/g of feces [IQR: −1.50 to −0.01]), showing a significant difference between the two groups (*n* = 8 each; *p* = 0.024) (Figure 5B). The change in fecal succinic acid concentration significantly discriminated between the RJ power-decreased and RJ power-increased groups, with an area under the curve (AUC) of 0.84 (logistic regression χ^2^ = 8.18, *p* = 0.004). No such differences were observed in the non-synbiotics group.

## 4. Discussion

This study demonstrated that the physical performance in judokas unexpectedly deteriorated before the match despite continued high-intensity training. However, with synbiotics intake, the deterioration of physical performance was relatively maintained from 4 weeks to 3 days before the match. In addition, in the synbiotics group, the change in fecal succinic acid concentration was negatively associated with the rate of change in physical performance, particularly in RJ power. In the RJ power-increased group, the change in fecal succinic acid concentration was significantly lower compared to that in the RJ power-decreased group. These results suggest that consuming synbiotics and reducing fecal succinic acid concentration before the match may help maintain physical performance in judokas.

Although high-intensity training is commonly performed before the match to improve athletic performance, physical performance unexpectedly declined from 4 weeks to 3 days before the match in the present study. One possible explanation for this finding is the effect of weight reduction undertaken in preparation for the match [20]. Previous studies have reported that rapid weight loss induces physiological changes such as energy deficiency, dehydration, and reductions in muscle glycogen, which may negatively affect physical performance [7,9,21,22,23]. However, these studies have primarily focused on rapid weight loss conducted in the few days immediately before the match. This is the first study to examine the changes in body composition and physical performance during the 4 weeks before the match in judokas. In addition, previous studies have suggested that the accumulation of fatigue associated with increased training load in the weeks before the match may lead to a decline in physical performance [24]. Furthermore, insufficient energy intake has been reported to impair recovery from fatigue and negatively affect training adaptation and physical performance [25]. High-intensity training under conditions of energy deficiency may impair adequate recovery. This can lead to fatigue accumulation and a subsequent decline in physical performance. Accordingly, it is necessary to identify appropriate management approaches that allow judokas to maintain physical performance during weight reduction before the match.

**Synbiotics intake appeared to attenuate the decline in physical performance from 4 weeks to 3 days before the match.** The most notable finding of the present study was that synbiotics intake relatively attenuated the decline in physical performance observed from 4 weeks to 3 days before the match. Recent studies have suggested that gut microbiota play an important role in host immune function, inflammatory responses, and energy metabolism [26,27]. Modulation of the gut environment mediated by changes in gut microbiota composition and microbial metabolites may contribute to maintaining athletes’ conditioning and performance [26,27,28]. In addition, previous studies have reported that the intake of probiotics or synbiotics can suppress inflammatory responses and facilitate recovery from exercise-induced fatigue [28,29,30,31,32]. Therefore, it is possible that synbiotics intake modulated the gut environment and reduced fatigue accumulation through the suppression of inflammatory responses, thereby attenuating the decline in physical performance before the match.

**In the synbiotics group, the change in fecal succinic acid concentration may be negatively associated with the rate of change in physical performance, particularly in RJ power. In the RJ power-increased group, the change in fecal succinic acid concentration was significantly lower compared to that in the RJ power-decreased group.** In the synbiotics group, the change in fecal succinic acid concentration was negatively associated with the rate of change in physical performance. These results suggest that reducing fecal succinic acid concentration may contribute to maintaining physical performance. In our previous study, we revealed that low fecal succinic acid concentration before the start of training may enhance the effects of T-HIIT on improvement in physical performance [11], which is not in conflict with the current findings. The change in fecal succinic acid concentration increased in the RJ power-decreased group, whereas it decreased in the RJ power-increased group, and the difference between the groups was statistically significant. Therefore, it is suggested that reducing fecal succinic acid concentration may be a crucial factor in maintaining and enhancing RJ power.

**The relevance of succinic acid as a biomarker.** Fecal succinic acid may be a useful non-invasive biomarker reflecting the metabolic state of the gut microbiota. Succinic acid is an important intermediate metabolite in metabolic pathways of both the host and gut bacteria, and its concentration in the intestinal tract is controlled by the balance between succinate-producing and consuming bacteria. In a healthy gut environment, succinic acid is rapidly consumed by propionic acid-producing bacteria and other organisms, and fecal succinic acid concentration is known to be kept low. On the other hand, if this metabolic pathway is disrupted, succinic acid accumulates, which is thought to reflect functional disruption or dysbiosis of the gut microbiota. Therefore, the fecal succinic acid concentration can serve as an indicator that comprehensively reflects the metabolism of the entire gut microbiota. Furthermore, succinic acid serves as a ligand of SUCNR1 (GPR91), which is expressed in immune cells, adipose tissue, and skeletal muscle, and has been reported to enhance inflammatory responses [33,34]. Therefore, a sustained increase in succinic acid may lead to a chronic low-inflammation state. From an exercise physiological perspective, such sustained activation of inflammatory signals can adversely affect skeletal muscle function. Thus, a chronic increase in gut-derived succinic acid may not only reflect changes in the gut environment but also contribute to decreased exercise capacity. It should be noted that the present study demonstrates an association rather than a causal relationship, and the proposed mechanisms remain hypothetical. Therefore, fecal succinic acid should be interpreted as a potential biomarker rather than a causal determinant of physical performance. Further mechanistic and interventional studies are required to clarify its functional role.

### Several Limitations

There are several limitations in this study. First, the number of study subjects was small (*n* = 16), and the subjects were from a single university judo club. This limits the statistical power and generalizability of the findings. Therefore, the results should be interpreted with caution. It may be necessary to further confirm the results of the current study with a larger sample size from multiple institutions.

Second, the relationship between changes in the gut environment due to synbiotics intake and pre-match conditioning could not be sufficiently clarified in this study. In particular, the mechanisms underlying the maintenance of physical performance during weight reduction associated with synbiotics intake were not elucidated. Further studies including detailed analyses of the gut microbiota and inflammation-related markers may help to better clarify the relationship between synbiotics intake and pre-match conditioning.

Third, although the fecal succinic acid concentration showed some association with the physical performance of judokas, the mechanism was not elucidated in this study. The impact of high fecal succinic acid concentrations on physical performance in judokas should be further investigated in future studies.

Fourth, this study employed a repeated-measures design to evaluate within-subject associations between synbiotics use and the maintenance of physical performance. Although a crossover design could further strengthen causal inference, such an approach was not feasible in the present match environment due to constraints related to the match schedule. Therefore, the findings of this study should be interpreted as associations rather than causal effects and should be further examined in future studies.

## 5. Conclusions

In conclusion, our study revealed that physical performance in judokas tends to deteriorate before the match, and taking synbiotics may have some potential to prevent this deterioration. In addition, the change in the fecal succinic acid concentration may be negatively associated with the rate of change in physical performance, particularly in RJ power. These results suggest that consuming synbiotics and reducing the fecal succinic acid concentration before the match may be associated with the maintenance of physical performance in judokas, which may have implications for match performance.

## Figures and Tables

**Table 1:** Characteristics of study participants.

Men (*n* = 16)		Non-Synbiotics Group	Synbiotics Group
**Age (years)**		18.9 ± 1.0	19.9 ± 1.0
**Height (cm)**		173.0 ± 4.8	173.1 ± 4.8
**Body weight (kg)**		81.8 ± 15.0	82.2 ± 14.0
**Muscle mass (kg)**		64.2 ± 8.6	63.5 ± 7.9
**Body fat (%)**		16.6 ± 4.1	17.9 ± 5.0
**Muscle mass/Fat amount ratio**		5.1 ± 1.4	4.8 ± 1.7
**Years of experience (year)**		12.7 ± 2.0	13.7 ± 2.0
**Judo rank (*n*)**	**2nd-degree black belt**	15	15
	**1st-degree black belt**	1	1

The values are the means and standard deviations.

## Data Availability

Data are available from the corresponding author upon reasonable request.
